# Functional EpoR Pathway Utilization Is Not Detected in Primary Tumor Cells Isolated from Human Breast, Non-Small Cell Lung, Colorectal, and Ovarian Tumor Tissues

**DOI:** 10.1371/journal.pone.0122149

**Published:** 2015-03-25

**Authors:** Scott D. Patterson, John M. Rossi, Katherine L. Paweletz, V. Dan Fitzpatrick, C. Glenn Begley, Leigh Busse, Steve Elliott, Ian McCaffery

**Affiliations:** 1 Department of Medical Sciences, Amgen Inc., Thousand Oaks, California, United States of America; 2 Department of Molecular Sciences, Amgen Inc., Thousand Oaks, California, United States of America; 3 Department of Oncology Research, Amgen Inc., Thousand Oaks, California, United States of America; Albert Einstein College of Medicine, UNITED STATES

## Abstract

Several clinical trials in oncology have reported increased mortality or disease progression associated with erythropoiesis-stimulating agents. One hypothesis proposes that erythropoiesis-stimulating agents directly stimulate tumor proliferation and/or survival through cell-surface receptors. To test this hypothesis and examine if human tumors utilize the erythropoietin receptor pathway, the response of tumor cells to human recombinant erythropoietin was investigated in disaggregated tumor cells obtained from 186 patients with colorectal, breast, lung, ovarian, head and neck, and other tumors. A cocktail of well characterized tumor growth factors (EGF, HGF, and IGF-1) were analyzed in parallel as a positive control to determine whether freshly-isolated tumor cells were able to respond to growth factor activation *ex vivo*. Exposing tumor cells to the growth factor cocktail resulted in stimulation of survival and proliferation pathways as measured by an increase in phosphorylation of the downstream signaling proteins AKT and ERK. In contrast, no activation by human recombinant erythropoietin was observed in isolated tumor cells. Though tumor samples exhibited a broad range of cell-surface expression of EGFR, c-Met, and IGF-1R, no cell-surface erythropoietin receptor was detected in tumor cells from the 186 tumors examined (by flow cytometry or Western blot). Erythropoiesis-stimulating agents did not act directly upon isolated tumor cells to stimulate pathways known to promote proliferation or survival of human tumor cells isolated from primary and metastatic tumor tissues.

## Introduction

Patients with cancer commonly develop anemia arising from the effect of the tumor itself or from treatments such as chemotherapy [1, 2]. Since anemia is an independent risk factor for mortality [3, 4], treating anemia with consideration of the associated risks remains important for the care of patients with cancer. Erythropoiesis-stimulating agents (ESAs) are recombinant glycoproteins that stimulate red-blood-cell production using the same molecular mechanism as endogenous erythropoietin [5]. Randomized, controlled trials have shown that ESA use in patients with cancer receiving chemotherapy reduces red-blood-cell transfusion requirements [6–8]. However, safety concerns have arisen around ESA use with various trials reporting that ESA administration promoted tumor progression and adversely impacted overall survival rates (trials reviewed in [9]).

The mechanism(s) by which ESAs might affect survival or stimulate tumor-cell growth is not clear. Indirect mechanisms have been proposed including (i) ESA-mediated promotion of thrombovascular events leading to increased mortality and (ii) ESA-mediated stimulation of angiogenesis leading to increased tumor growth (10) although recent results do not support this mechanism [11, 12]. An alternative hypothesis is that ESAs may directly increase tumor cell proliferation and survival by activating EpoR on tumor cells [13, 14].

Studies examining the hypothesis that ESAs directly stimulate tumor growth by activating canonical EpoR signaling pathways (i.e. PI3K/AKT, RAS/RAF/ERK and JAK/STAT) on tumor cells have reported conflicting results. Several studies reported that primary tumor tissue and tumor-derived cell-lines express *EpoR* mRNA transcript and contain EpoR protein as shown by Western blot analysis or immunohistochemistry (IHC) [15–17]. A possible confounding variable in these studies is that mRNA analysis of bulk tumor tissue includes representation of stromal cells and other infiltrating cell types from blood. Also, the quantities of *EpoR* mRNA detected in some tumor and normal cells outside the erythroid compartment are relatively low (at levels10- to 1000-fold lower than in positive controls) and calls into question whether these mRNA levels are adequate to produce relevant amounts of functional EpoR protein [12, 18–20].

Many studies employing Western blotting and IHC often used commercially available polyclonal EpoR antibodies that have been shown to lack EpoR specificity [21, 22]. Importantly, these studies could not address erythropoietin-dependent EpoR function in tumor tissue. More recent, detailed studies have reported that tumor cell-lines, tumor biopsies, and endothelial cells did not contain increased levels of *EpoR* mRNA, or protein compared with normal tissues [23–25] and that there was no amplification of the *EpoR* gene in tumor cells [23]. Additionally, xenograft models were conducted using a limited number of breast cell-lines that suggested co-administration of rHuEpo resulted in diminished efficacy of Her2 directed agents in Her2+ cell-lines [26]. In contrast, several studies using tumor cell-lines showed an improved tumor response with administration of ESAs [27, 28]. These results demonstrate the continuing challenges in the field that confound clear conclusions to be drawn regarding the ESA tumor stimulation hypothesis. However, as methods to study signaling in freshly-derived human tumor cells have only recently become available, the literature has mostly relied on cell-lines whose relevance is uncertain.

It has been hypothesized that ESAs are able to directly stimulate tumor cells. This study was performed to specifically address this hypothesis. EpoR pathway activation was analyzed on viable human tumor cells obtained directly from human tumor tissues representing a range of different primary tumor types. This included evaluation of receptor function and protein expression in freshly-derived human breast tumor tissues, including both Her2+ and Her2- tumors to address specific questions related to the biological relevance EpoR in breast cancer. We examined if ESA exposure could activate signaling pathways by treating viable primary human tumor cell isolates with recombinant human erythropoietin (rHuEpo) and analyzing the effect on the activation state of multiple signaling proteins downstream of cell-surface receptors. Cell-surface expression of EpoR, as well as total EpoR (assessed in breast cancer sample cohort) was also analyzed using specific EpoR monoclonal antibodies [11].

## Materials and Methods

### Cell Culture

The megakaryoblastic leukemia cell-line, UT-7/Epo [29] was a gift from Dr. Norio Komatsu, Jichi Medical School, Minamikawachi, Japan. The colorectal adenocarcinoma cell-line HT29 was purchased from ATCC (Rockville, MD). Although not formally authenticated, control cell-line performance was consistent over the duration of the study, with no aberrant changes observed with regards to receptor level expression and response to cytokines as measured in flow cytometry experiments. Prior to growth factor stimulation, HT29 and UT-7/Epo were starved overnight in media containing 0.1% (w/v) bovine serum albumin (BSA). UT7/Epo and HT29 cells were harvested and washed by centrifugation twice with Ca^2+^/Mg^2+^ free phosphate-buffered saline (PBS; Invitrogen).

Aqua Viability Reagent (Invitrogen) was added per the manufacturer’s protocol for exclusion of dead cells. Cell densities were adjusted to 10^6^ viable cells/mL prior to growth factor stimulation. For additional cell culture conditions, see S1 Appendix.

### Erythroid Progenitor-Cell (EPC) Assay

Human CD34+ progenitor cells (AllCells Inc., Emeryville, CA) were isolated from bone marrow using CD34 immunomagnetic purification (Miltenyi Biotec, Auburn, CA), per the manufacturer’s protocol. Differentiation of EPCs was induced with rHuEpo (0.1 U/mL), IL-3, IL-6, and stem cell factor (SCF) (R&D Systems, Minneapolis, MN). The use of CD36+/CD34- expression as markers for erythroid lineage development has been previously described [19, 30]. EpoR function was analyzed by exposing the culture at various time points to a range of rHuEpo concentrations from 0 U/mL (rHuEpo formulation buffer “vehicle” control: 100 mM NaCl, 20 mM NaCitrate, 0.25% human serum albumin [HAS], pH 6.9) to 300 U/mL for 5 or 30 minutes, which includes the physiological range of endogenous Epo (5–30 mU/mL) and exceeds the pharmacological levels observed in patients treated with ESAs (reported to be a mean of 1 U/mL in plasma) [31]. Thus, the upper range of the titration was at least 300-fold higher than the theoretical exposure of tumor cells in patients administered ESAs. EpoR cell-surface expression was determined by flow cytometry using the EpoR specific antibody MAb307 (R&D Systems) incorporating 7-amino-actinomycin-D (7-AAD; Invitrogen) staining to select live cells with intact plasma membranes.

### Tissue Processing

Samples were obtained from Asterand USA, BIO OPTIONS, Inc, and the MT Group. Tumor content was characterized by: 1) A qualified, independent pathologist (all samples included in this study were confirmed to be tumors). 2) H&E staining (tumor content ranged from 6% to 100%; S1 Fig.). 3) DNA content (median tumor aneuploidy percentage, interquartile range, was 56%; S1 Table and S1 Appendix). 4) pERK and pAKT induction of 19 matched normal colon and tumor samples stimulated by growth factors (tumor samples had higher pathway induction than normal colon samples; S2 Fig. and S1 Appendix). In addition, flow cytometry experiments were designed to isolate an equivalent number of viable tumor cells from each sample (see details in the next section).

For disaggregation, human tumor tissues were digested with 0.34 U/mL dispase (Roche Diagnostics, Indianapolis, IN) and 1mg/mL DNAse (Worthington Corp., Lakewood, NJ) for 30 minutes at 37°C. Cell suspensions were washed, cell viability and density determined, and cell densities adjusted to 10^6^ viable cells/mL in medium. Aqua Viability Reagent was added to cell suspension per manufacturer’s protocol to label dead cells.

### Effect of Growth Factor and rHuEpo Addition

Aqua Viability Dye-labeled cell-lines and tumors were stimulated for 5 or 30 minutes with vehicle, a serial dilution of rHuEpo (vehicle to 300U/mL), or a cocktail of epidermal growth factor (EGF)/hepatocyte growth factor (HGF)/insulin-like growth factor-1 (IGF-1) (EGF: 100 ng/ml, Roche, Basel, Switzerland; HGF: 200 ng/ml, IGF-1: 100 ng/ml, R&D Systems) for 5 and 30 minutes in aliquots of 10^6^ cells. Treated cells were fixed by adding an equal volume of pre-warmed (37°C) fix buffer I (BD Biosciences) and incubated for 10 minutes at 37°C. Fixed cells were then permeabilized in ice-cold 90% (v/v) methanol and stored at -20°C prior to flow cytometry analysis.

### Analysis of Intracellular Signaling by Flow Cytometry

Fixed and permeabilized cells were washed twice with ice-cold Fluorescence-Activated Cell Sorter (FACS) Stain Buffer (2% FBS [v/v], 0.09% [w/v] NaN_3_; BD Biosciences). Samples were stained for 1 hour at room temperature with fluorochrome-conjugated antibodies specific for phosphorylated forms of AKT (AF-647; Cell Signaling Technology, Danvers, MA), ERK1/2 (AF-647; Cell Signaling Technology), and STAT5 (AF-488; BD Biosciences). EpCAM (PerCP-Cy5.5; BD Biosciences), pan-cytokeratin (PE; BD Biosciences), and active Caspase-3 (AF-405; Cell Signaling Technology) were multiplexed with phospho-specific antibodies listed above to allow for gating of viable, non-apoptotic epithelial cells. Stained samples were run on an LSR II (Becton Dickinson, Franklin Lakes, NJ) with 10^4^ viable epithelial events acquired. The analysis used a gating strategy for viable EpCAM or cytokeratin positive events excluding active caspase-3 (apoptotic) or Aqua Viability dye-positive events. Results are reported as mean fluorescence intensity (MFI) fold change in treated samples compared to vehicle.

### Analysis of Cell-surface Receptors in Live Cells by Flow Cytometry

Tumor cells and control cell-lines were stained with antibody cocktails (see S1 Appendix for the antibodies used). Prior to flow cytometry, samples were stained with 5 μg/mL 7-AAD to exclude dead and apoptotic cells. Flow cytometry was performed using an LSR II (BD Biosciences). Receptor levels were reported as a ratio of MFI values relative to the appropriate isotype control. The analysis employed a cell-gating strategy that selected viable EpCAM positive cells and excluded CD45-positive and dead/apoptotic cells. For each sample, 10^4^ viable EpCAM positive events were acquired.

### Analysis of EpoR Protein Expression by Western Blot

Tumor tissues were homogenized by sonication on ice in a lysis buffer containing 50 mM Tris-HCl, pH 7.2, 150 mM NaCl, 1% Triton X (v/v), 0.1% Na deoxycholate (w/v) and a protease inhibitor cocktail (0.1 mg/mL 4-(2-aminoethyl) benzenesulfonyl fluoride hydrochloride [Pefabloc-SC], and 10 mg/mL pepstatin). Lysates from cell-line controls were prepared in the same manner. Lysates were subjected to SDS-PAGE (NuPAGE; Invitrogen) and transferred to Invitrolon polyvinylidene fluoride membranes (Invitrogen) and processed as described in the S1 Appendix.

## Results

### EpoR Pathway Activation in an EPC Assay

**Fig 1:**
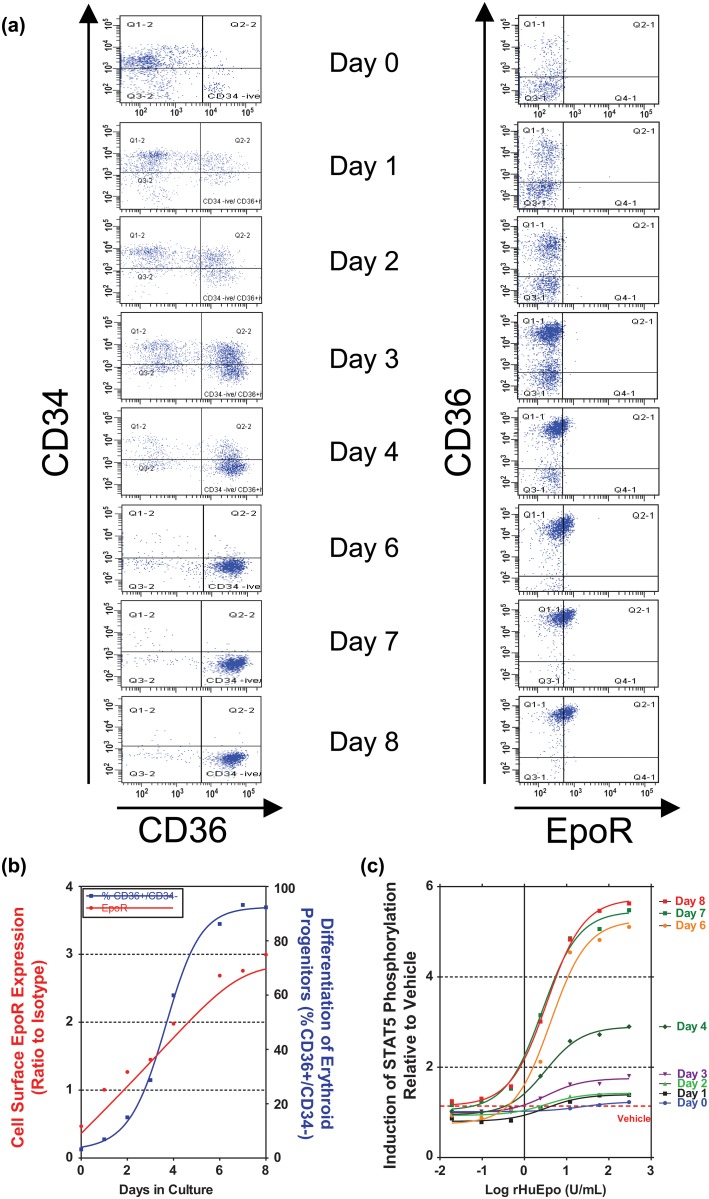
Characterization of cell-surface EpoR expression and function in differentiating primary erythroid progenitors (EPCs). (A) Analysis of the profile of EPC differentiation over the course of the 8-day culture. (B) Analysis of the relationship between differentiation of EPCs and EpoR expression. The % of total cells in the culture that were CD36+/CD34- is shown. EpoR levels are reported as a ratio of mean fluorescent intensity (MFI) values relative to the appropriate isotype control. (Note: the ratio to isotype was used to normalize data and does not serve as baseline measure; i.e., a ratio of 1 does not imply a lack of receptor expression). (C) At each time-point, EPCs were harvested and stimulated with a range of rHuEpo (0 U/mL [vehicle] up to 300U/mL) to evaluate EpoR function. The MFI of pSTAT5 at each rHuEpo concentration is expressed as a ratio relative to vehicle treated cells.

To define assay sensitivity and specificity, EpoR function and cell-surface expression was evaluated during 8-day cultures of a normal, physiologically relevant Epo-responsive tissue, differentiating primary human EPCs. CD34+ cells were isolated from human bone marrow and exposed to SCF, IL-3, IL-6, and a low concentration of rHuEpo (0.1 U/mL). During the time course, EPC numbers increased as shown by the accumulation of the CD36+/CD34- cells from the CD34+ enriched population (Fig. 1A). Cell-surface EpoR expression was analyzed by flow cytometry using the MAb307 antibody. Levels of EpoR expression increased from undetectable levels on day 0 to maximum levels by day 8 (Fig. 1B).

At each time point, a range of rHuEpo (0 U/mL [vehicle] up to 300 U/mL) was added to EPCs. Levels of phospho-proteins (pSTAT5, pAKT, and pERK) known to be induced by activated EpoR were measured by flow cytometry. EpoR function was determined by comparing levels of each phospho-protein in the rHuEpo-treated cells relative to the vehicle treatment. Similar approaches have been reported in the literature [19, 32, 33]. As shown in Fig. 1C, no rHuEpo-dependent signaling was observed on day 0. Dose-dependent increases in pSTAT5 were observed on day 1 (Fig. 1C) when only 6.9% of cultured cells were CD36+ (Fig. 1A) and low levels of cell-surface EpoR were observed (Fig. 1B). At each of these time-points, concentrations of rHuEpo above 10 U/mL stimulated pSTAT5 to maximal levels and no further induction was observed at higher concentrations of rHuEpo. By day 8, when > 90% of the culture had an erythroid phenotype and were EpoR positive, significant levels of pSTAT5 were observed at rHuEpo concentrations as low as 0.02 U/mL (Fig. 1C). Representative histograms demonstrating rHuEpo driven pSTAT5 induction are shown in S3 Fig. Similar data were observed for pAKT and pERK (data not shown).

The sensitivity and specificity of the EPC assay ensured that EpoR function could be examined in primary cells exposed to rHuEpo levels that are physiologically relevant (range 0.005 U/mL to 0.03 U/mL) and approximately 30-fold lower than maximal concentrations of serum ESA observed in patients treated with ESAs [31].

**Fig 2:**
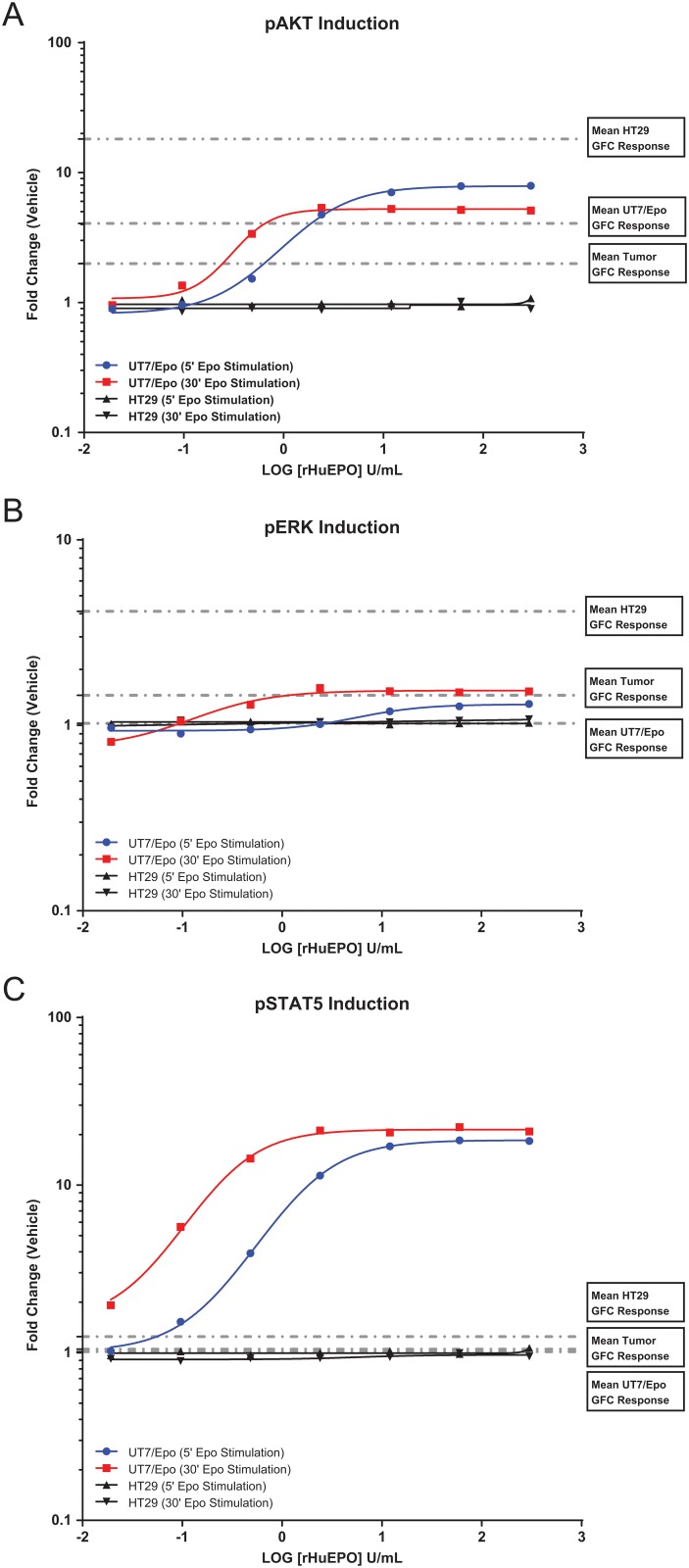
rHuEpo dose response curves for control cell-lines UT-7/Epo and HT29 at 5 and 30 minute stimulation time points. Dotted lines indicate mean fold change values for growth factor stimulation (i.e. EGF/HGF/IGF1). HT29 (N = 38), UT-7/Epo (N = 38), or disaggregated human tumors (N = 181). (A) pAKT induction. For UT-7 Epo cells, the mean (± standard deviation) pAKT induction was 3.68 (±1.17) for the growth factor cocktail and 8.24 (±1.98) for Epo. For HT29 cells, the mean (± standard deviation) pAKT induction was 16.64 (±4.70) for the growth factor cocktail and 0.95 (±0.13) for Epo. (B) pERK induction. For UT-7 Epo cells, the mean (± standard deviation) pERK induction was 1.02 (±0.11) for the growth factor cocktail and 1.38 (±0.29) for Epo. For HT29 cells, the mean (± standard deviation) pERK induction was 4.12 (±0.98) for the growth factor cocktail and 1.00 (±0.07) for Epo. (C) pSTAT5 induction. For UT-7 Epo cells, the mean (± standard deviation) pSTAT5 induction was 1.02 (±0.07) for the growth factor cocktail and 16.21 (±6.56) for Epo. For HT29 cells, the mean (± standard deviation) pSTAT5 induction was 1.25 (±0.12) for the growth factor cocktail and 1.01 (±0.06) for Epo. Levels are reported as a ratio of mean fluorescent intensity (MFI) values relative to the appropriate isotype control. (Note: the ratio to isotype was used to normalize data and does not serve as baseline measure; i.e., a ratio of 1 does not imply a lack of receptor expression).

rHuEpo dose response was also evaluated in the positive control cell-line UT-7/Epo and the negative control cell-line HT29. UT-7/Epo is a known Epo-responsive cell-line and demonstrates pathway responsiveness along PI3K/AKT, RAS/RAF/ERK and the JAK/STAT signaling pathways. HT29 cells do not express detectable levels of EpoR and are not responsive to rHuEpo [28] and thus serve as a negative control for rHuEpo addition. In UT-7/Epo cells, maximum pSTAT5, pAKT, and pERK induction levels were reached by 10 U/mL rHuEpo and no further induction was seen in concentrations up to 300 U/mL rHuEpo (Fig. 2). In HT29 cells, no induction was observed across all rHuEpo concentrations. For every human tumor that was analyzed for response to rHuEpo and also for EpoR expression, both UT7/Epo and HT29 cell-lines were processed in parallel as experimental controls. Representative histograms demonstrating pSTAT5, pAKT, and pERK induction in the UT7/Epo cell-line are shown in S4 Fig.

**Fig 3:**
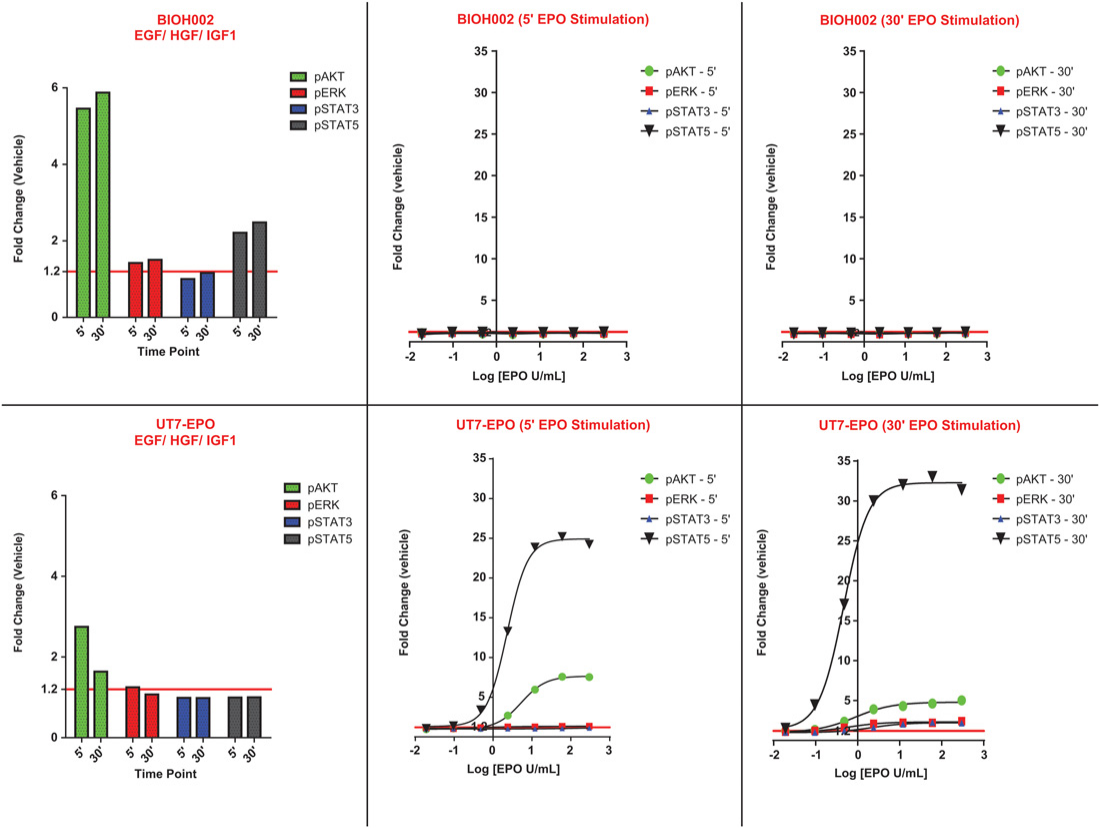
rHuEpo dose response curves for the control cell-line UT-7/Epo and a representative tumor sample BIOH002 (head-and-neck cancer) at 5 and 30 minute stimulation time points. Levels are reported as a ratio of mean fluorescent intensity (MFI) values relative to the appropriate isotype control. (Note: the ratio to isotype was used to normalize data and does not serve as baseline measure; i.e., a ratio of 1 does not imply a lack of receptor expression).

A representative tumor sample from head-and-neck cancer (BIOH002) is shown in Fig. 3. In contrast to the Epo responsive UT-7/Epo cell-line, no pSTAT5, pAKT, and pERK induction was observed at any rHuEpo concentration. A separate pool of cells isolated from the head-and-neck cancer biopsy were stimulated with a growth factor cocktail of EGF/ HGF/IGF1 and demonstrated response along pAKT, pERK and pSTAT5 pathways. The growth factor cocktail response served as a positive control and clearly demonstrated that tumor cells isolated from this patient sample responded to known tumor growth factors in a robust and measurable fashion.

### EpoR Pathway Activation in Tumor Cells Isolated From Human Tumor Tissues

To evaluate EpoR function in tumors, tissues were obtained from surgical resections (using IRB-approved protocols) from various tumor types, stages, and grades (Table 1). All tumors, except where indicated, were collected from patients not previously treated with chemotherapy. A number of metastatic tissues were also included to determine whether response to rHuEpo was altered during disease progression.

**Table 1:** Distribution and Histological Details of Tumor Types Analyzed.

	Colon	Breast	Lung	Ovarian	Head and Neck	Kidney	Pancreas	Prostate	Cervix	Gastric
Total number of pts	48	38	44	35	5	4	2	1	1	3
Adenocarcinoma	48	38	22	11	2	0	2	1	1	3
Squamous carcinoma	0	0	17	0	3	0	0	0	0	0
Other	0	0	5^a^	24^b^	0	2^c^	0	0	0	0
Pts with locally recurrent disease	1	1	1	12	0	0	0	0	0	0
Pts with metastatic disease (nodal)	16	22	9	11	4	0	0	0	0	2
Pts with metastatic disease (distant)	9	1	0	6	1	1	1	0	0	1
Pts receiving prior chemotherapy	1	3	1	2	0	0	0	0	0	0
Stage I	1	1	25	7	0	3	0	0	0	0
Stage II	25	27	6	3	0	0	1	1	1	0
Stage III	16	7	7	13	2	1	0	0	0	1
Stage IV	3	1	0	2	0	0	0	0	0	2
NOS	3	2	6	10	3	0	1	0	0	0
Grade I	2	0	3	2	0	1	0	0	1	0
Grade II	26	8	15	0	2	0	0	0	0	0
Grade III	14	29	20	18	1	1	0	0	0	2
Grade IV	1	0	0	0	0	0	0	0	0	0
NOS	5	1	6	15	2	2	2	1	0	1
Metastatic tissues	4	4	1	2	0	0	1	0	0	1

Abbreviations: pts, patients; NOS, not otherwise specified

^a^Other lung tumors: large cell (2), carcinoid (2), carcinosarcoma (1);

^b^Other ovarian tumors: Brenner tumor (1), Mullerian mixed tumor of the ovary (2), Serous Papillary (7), Signet Ring Carcinoma (1), NOS (7), carcinosarcoma (1), clear cell (3), dysgerminoma (1);

^c^Other kidney tumors: clear cell (2).

Disaggregation of tumor tissue to obtain single-cell populations of both tumor and stromal compartments requires proteolytic enzymes which may compromise functional analysis of cell-surface receptors. To address this concern, dispase was used because it has a narrow range of specificities. In the EpoR dependent UT-7/Epo cell-line, no effect of dispase on EpoR function or expression was observed below 0.5 U/mL dispase compared with undigested cultures (S5 Fig.). Similarly, in the colorectal cancer cell-line HT29, no effect on EGFR, c-MET or IGF-1R function or expression was observed below 0.5U/mL dispase (data not shown). A concentration of 0.34 U/mL dispase was selected for tissue disaggregation as this amount had no observable effect on EpoR function in the UT7/Epo cell-line control model and also no effect on EGFR, c-MET or IGF-1R function in HT29 cells.

Single-cell suspensions from the disaggregated tumors were stimulated with a range of rHuEpo (0 to 300 U/mL) for 5 and 30 minutes. While maximal effects of rHuEpo are observed at ~ 1 U/ml, concentrations up to 300 U/mL were used to account for the possibility that tumor cells are less responsive to rHuEpo than EPCs. Early and late time points accounted for differing kinetics of PI3K/AKT, RAS/RAF/ERK, and JAK/STAT pathway induction observed in control cell-lines. Epo driven STAT5 phosphorylation was specifically targeted in this study because unlike PI3K/AKT, RAS/RAF/ERK, activation of STAT5 is more specific to the EpoR pathway and therefore provides a good assessment of specific EpoR activation. As a positive control to demonstrate that tumor cells were viable and responsive, they were stimulated with a cocktail of known tumor growth factors consisting of EGF, HGF, and IGF-1. Following addition of rHuEpo or the growth factor cocktail, cells were fixed, permeabilized, and analyzed by flow cytometry using a panel of fluorescently conjugated antibodies specific for pAKT and pERK, which are induced when EpoR, EGFR, c-Met, and IGF-1R are activated. pSTAT5 was included as it is tightly regulated by EpoR in EPCs.

**Fig 4:**
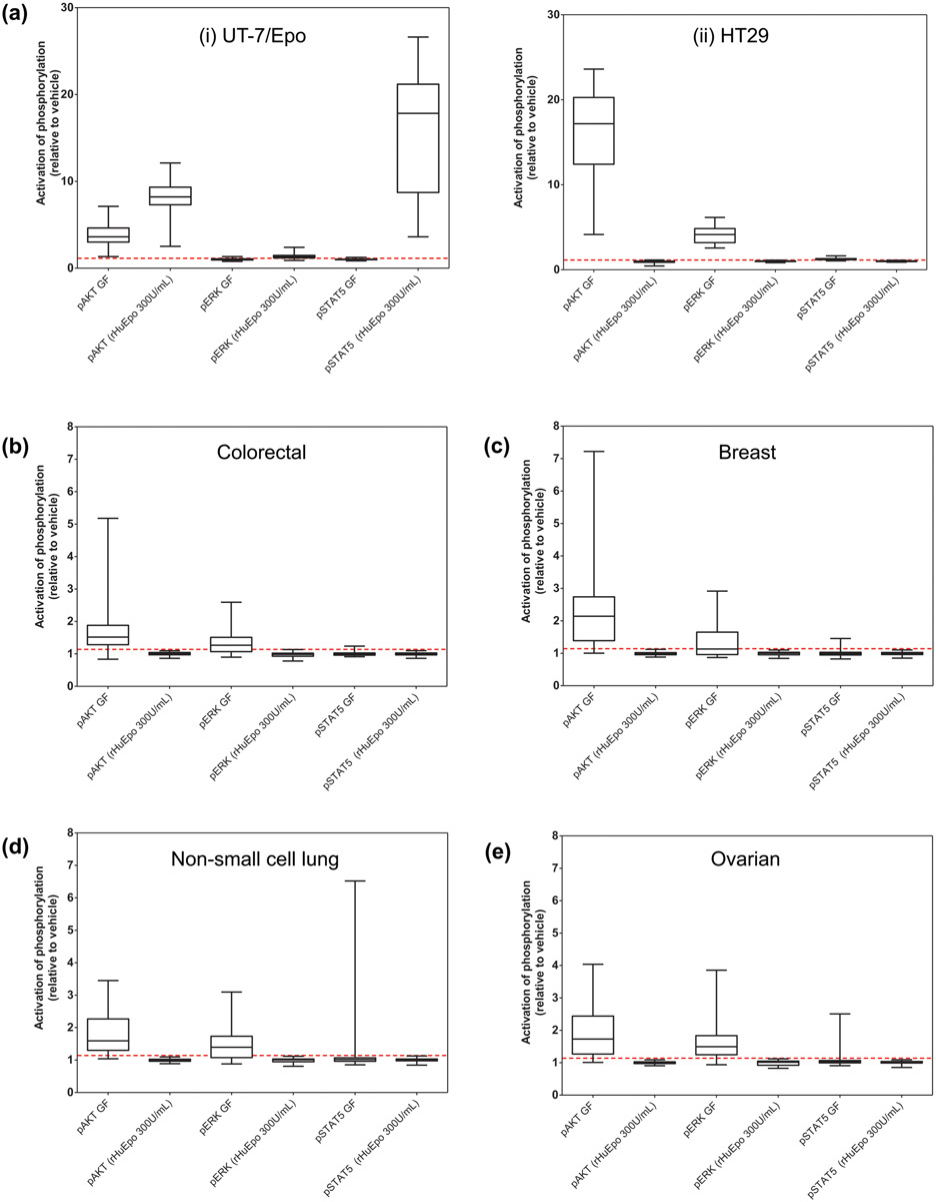
IGF-1R, c-Met, and EGFR but not EpoR are functional in primary tumor cell populations from human tumor tissues. Disaggregated tumors were stimulated with a range of concentrations of rHuEpo and growth factor controls for 5 and 30 minutes. These time points were chosen based upon experiments in the UT-7/Epo cell-line showing that phosphorylation peaked between 15 and 30 minutes for the majority of signaling proteins assayed (S7 Fig.). In disaggregated tumors, identical data were obtained for 5 and 30 minutes; therefore only the 5-minute data are shown. On each plot, the dotted line represents the threshold for pAKT (1.14) and pERK (1.16). Data shown for (A) cell-line controls (i) UT-7/Epo (ii) HT29 (n = 38 each cell-line) and tumors from (B) colorectal (n = 45), (C) breast (n = 34), (D) non-small cell lung (n = 43), (E) ovarian (n = 31). Levels are reported as a ratio of mean fluorescent intensity (MFI) values relative to the appropriate isotype control. (Note: the ratio to isotype was used to normalize data and does not serve as baseline measure; i.e., a ratio of 1 does not imply a lack of receptor expression).

To ensure that a lack of response was not simply due to inactive preparations of rHuEpo and to also verify potential false-positive or false negative events, signaling in HT29 and UT-7/Epo was analyzed in parallel with every tumor cell preparation. HT29 was selected as a positive control because it is known to express EGFR, c-Met, and IGF-1R, and is responsive to EGF, HGF, and IGF-1, activating the PI3K/AKT and RAS/RAF/ERK signaling pathways. These cells also served as a negative control for the rHuEpo addition. UT-7/Epo is a known Epo-responsive cell-line and served as a positive control. Both control cell-lines performed as expected and the observed effects were highly reproducible (Fig. 4A). Representative histograms showing pathway response to 30 minute stimulation with vehicle, 300 U/mL rHuEpo, or growth factor cocktail are depicted in S6 Fig.

Viable, non-apoptotic tumor cells were analyzed through the use of antibodies specific for epithelial tumor cell markers (EpCAM and pan-cytokeratin), a viability dye (debris exclusion), and an activated-caspase-3 antibody. These analyses provided a sensitive and specific method to characterize Epo-dependent effects on signaling pathways that are known to be critical for the growth and survival of tumor cells. The use of the growth-factor cocktail positive control also allowed comparisons with known tumor growth factors.

Stimulation of pAKT or pERK was observed in response to growth factors in tumor cells from colorectal (Fig. 4B; n = 48 patients), breast (Fig. 4C; n = 38 patients), NSCLC (Fig. 4D; n = 45 patients), and ovarian (Fig. 4E; n = 37 patients). This pathway-activation was the positive control and activation of at least one pathway was evident in each cell sample (although activation of all pathways was not always seen). In contrast, no rHuEpo-mediated activation of pERK, pAKT, or the canonical EpoR pathway constituent, pSTAT5, was observed in any of the tumor samples that were analyzed at any concentration of rHuEpo (the highest concentration employed, 300 U/mL, is represented). Similarly, no rHuEpo-mediated activation of pSTAT3 was observed in any of the tumor samples (S8 Fig.). In each experiment robust phosphorylation of STAT5 was observed with UT-7/Epo cells indicating that EpoR was specifically activated under the conditions used.

**Fig 5:**
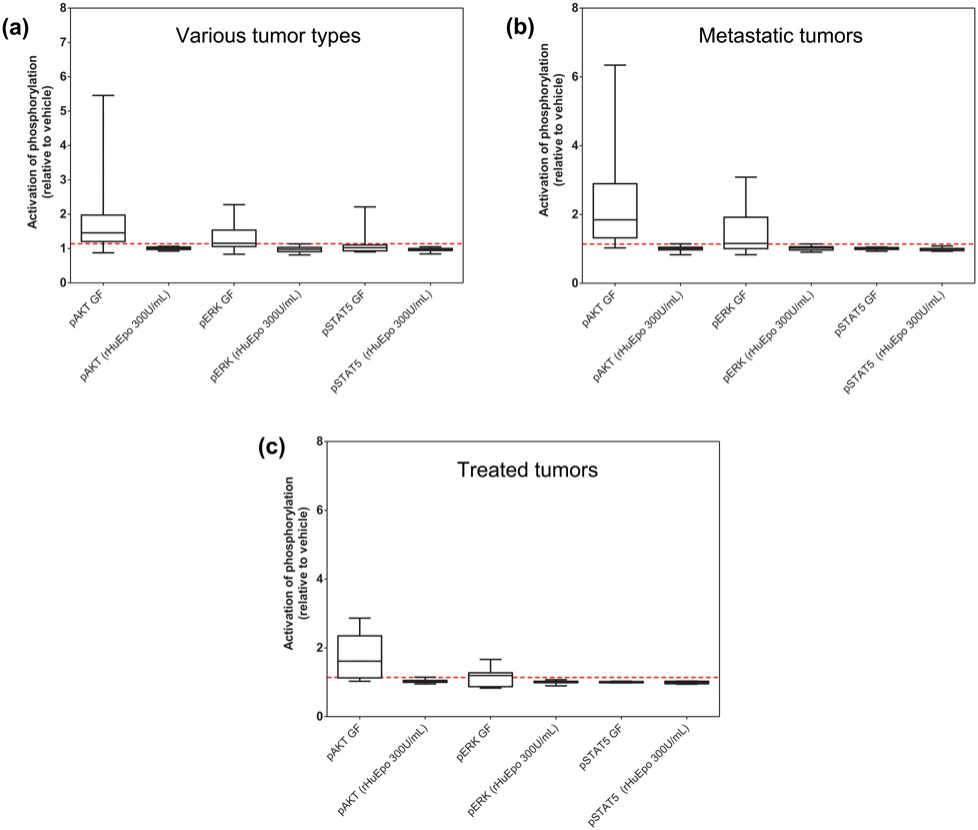
IGF-1R, c-Met, and EGFR but not EpoR are functional in freshly-derived tumor cell populations from human tumor tissues. Disaggregated tumors were stimulated with a range of concentrations of rHuEpo and growth factor controls for 5 and 30 minutes. (Identical data were obtained for 5 and 30 minutes; therefore only the 5-minute data are shown). The effect of rHuEpo or growth factor-cocktail addition on phosphorylation levels was expressed as the ratio of MFIs relative to vehicle-treated cells. On each plot, the dotted line represents the threshold for pAKT (1.14) and pERK (1.16). Data shown for tumors from (A) head and neck (n = 5), cervical (n = 1), kidney (n = 4), pancreas (n = 1), (B) metastatic tissues (colon metastatic to liver [n = 2], ovary [n = 1], lung [n = 1], pancreatic metastatic to liver [n = 1]; local nodal metastases from breast [n = 4], lung [n = 1], ovarian [n = 2]); (C) tumors from pre-treated patients (colorectal treated with oxaliplatin and bevacizumab [n = 1], breast treated with cyclophosphamide, methotrexate, fluorouracil [n = 2], breast treated with radiation, taxotere, cytoxan, taxol, [4 cycles each; n = 1], non-small cell lung treated with radiation [n = 2], ovarian treated with carboplatin and taxol [n = 2]).

Identical observations were made from head and neck (n = 5), kidney (n = 4), pancreatic (n = 2), cervical (n = 1), and gastric tumors (n = 3) (Fig. 5A) with no response to rHuEpo detected across a broad range of epithelial tumors types. As shown in Fig. 5B, there was no response in cells from metastatic lesions. Similarly, in samples derived from patients who had undergone various chemotherapeutic regimes there was no response to rHuEpo (Fig. 5C) although all displayed pathway-activation in response to the growth factors. Together these findings suggest that while tumor cells did respond to known epithelial-growth factors, they did not respond to rHuEpo in the original tumors, in metastatic lesions, nor following anti-cancer treatments.

In interpreting these data, it is critical to understand what represents a significant induction of phosphorylation as represented by MFI. To evaluate the variability of repeated measures of replicate vehicle-treated tumor cells, disaggregated cells from 10 tumor tissues were treated with vehicle in replicate (n = 10), and the ratio of the upper 95% CI of each MFI relative to the mean calculated. The ratios determined had a mean of 1.14 (range 1.07–1.15) for pAKT and a mean of 1.16 (range 1.08 to 1.22) for pERK. Therefore, a significant increase was defined as a response above a threshold ratio of 1.14 for pAKT and 1.16 for pERK. In principle, small numbers of tumor cells in the population could respond to rHuEpo and not be represented in MFI. To determine the sensitivity of the assays in terms of percentage of tumor cells at the selected thresholds for pAKT and pERK, mixing experiments using growth factor-stimulated and vehicle-treated populations were performed. The data demonstrate that a population MFI ratio relative to vehicle of 1.14 for pAKT and 1.16 for pERK would correspond to approximately 1% to 2% of responding tumor cells, suggesting that the method employed would be capable of detecting even a small sub-population of responsive cells in the rHuEpo-treated samples (data not shown). To further validate this, visual inspection of flow cytometry data was carried out for all rHuEpo-treated cells compared to the corresponding vehicle control. This rigorous exercise revealed no instance of activation of any sub-population of cells in rHuEpo-treated tumors, either in the tumor cell compartment or among the viable, non-apoptotic, non-epithelial stromal compartment of the tumor tissues.

### EpoR Protein Expression in Tumor Cells Isolated From Human Tumor Tissues

To support the lack of Epo driven pathway activation observed in disaggregated tumor samples, cell-surface EpoR was also analyzed in disaggregated, live tumor cells from the cohort of 186 patients (Table 1) by flow cytometry using the specific anti-EpoR antibody MAb307. This antibody was previously validated as specific by immunoprecipitation followed by Western blot using the A82 antibody [11] and by flow cytometry with live cells [19]. However, in other experiments it was observed that MAb307 cannot be used directly for Western Blot or IHC because of lack of detection of unfolded EpoR [34]. The specificity of MAb307 when used for flow cytometry was demonstrated using the EpoR-expressing positive control cell-line UT-7/Epo and the EpoR-negative control cell-line HT29. It is important to note that in this study, EpoR expression was evaluated by flow cytometry on the extracellular membrane of viable cells. Apoptotic cells and debris generated during the disaggregation process were excluded from analysis to avoid false positive EpoR detection. Under these conditions MAb307 was shown to be specific for cell surface EpoR in positive control cell-lines. In EpoR negative cell-lines, MAb307 MFI signal intensity was equivalent to a matched isotype control. MAb307 had similar sensitivity and specificity for EpoR as the A82 antibody (S9 Fig.) [11]. The membrane-impermanent DNA stain 7-AAD was used to select intact, viable cells thereby excluding contributions from functionally irrelevant intracellular EpoR. Additionally, an anti-EpCAM antibody was used to identify epithelial tumor cells in each disaggregated tissue. Levels of EGFR, c-Met, and IGF-1R were also measured using specific antibodies.

**Fig 6:**
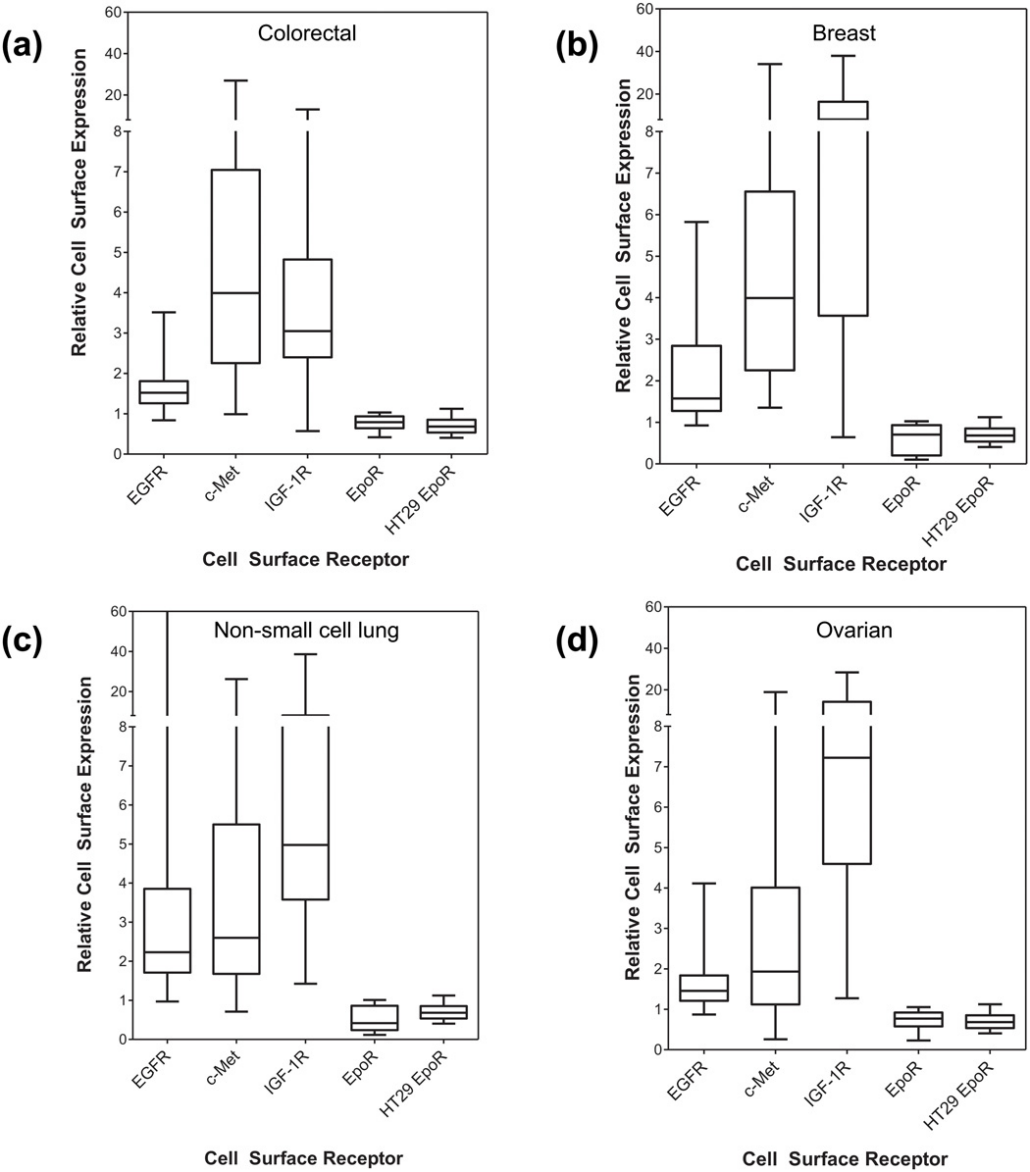
Levels of EpoR, IGF-1R, c-Met, and EGFR in freshly-derived tumor cell populations from human tumor tissues. Tumors were disaggregated with dispase (0.34U/mL) and EpoR levels determined by flow cytometry. Cell-surface receptor levels are expressed as a ratio to the appropriate isotype control to allow comparison of relative levels of receptor between tissues. On each plot, the negative EpoR control cell-line (HT29) is shown for comparison (n = 44 determinations). Data shown for tumors from (a) colorectal (n = 46), (b) breast (n = 34), (c) non-small cell lung (n = 41), (d) ovarian (n = 35).

**Fig 7:**
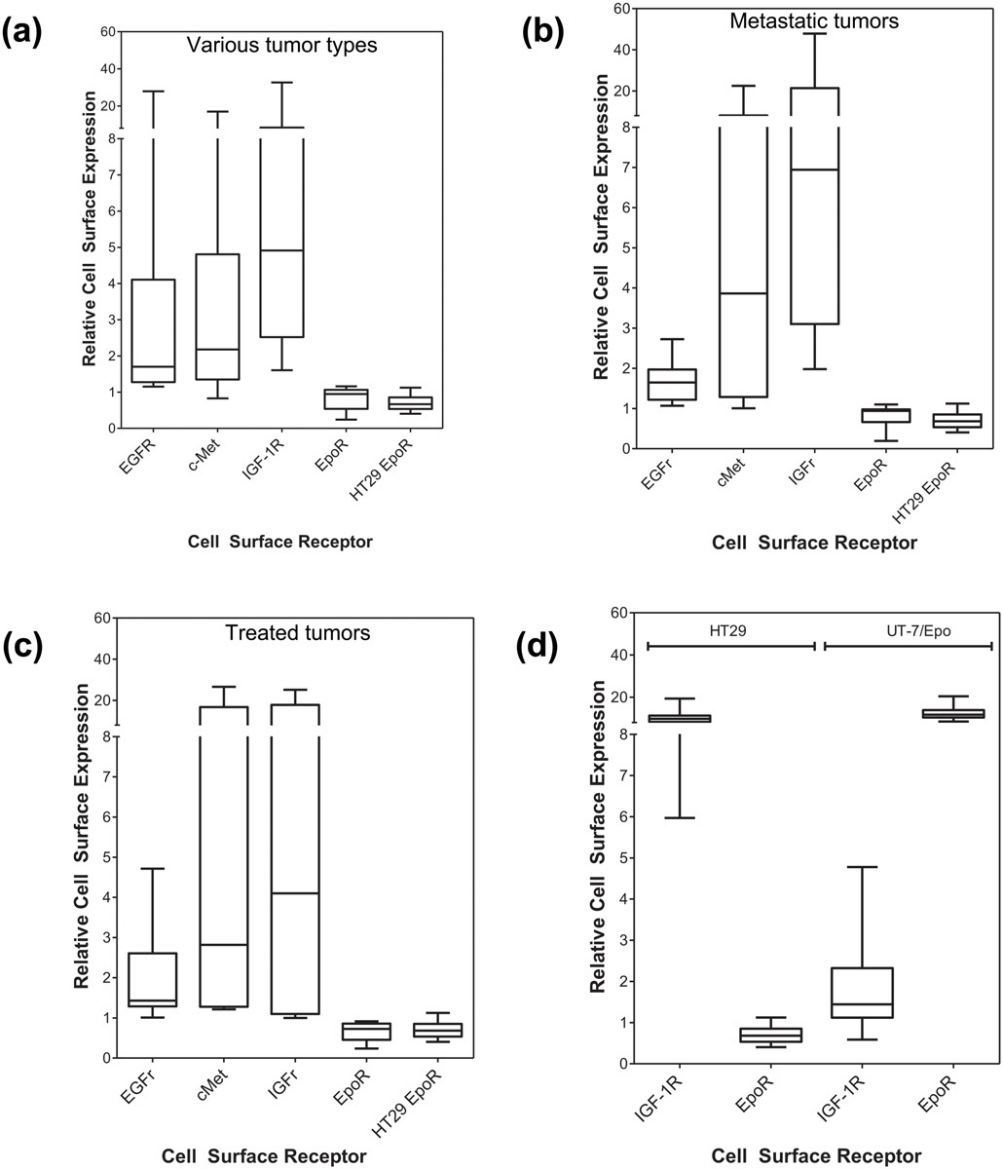
Levels of EpoR, IGF-1R, c-Met, and EGFR in freshly-derived tumor cell populations from human tumor tissues. Tumors were disaggregated with dispase (0.34U/mL) and EpoR levels determined by flow cytometry. Cell-surface receptor levels are expressed as a ratio to the appropriate isotype control to allow comparison of relative levels of receptor between tissues. On each plot, the negative EpoR control cell-line (HT29) is shown for comparison (n = 44 determinations). Data shown for tumors from (A) head and neck (n = 5), gastric (n = 2), kidney (n = 3), liver (n = 1), pancreas (n = 1), esophagus (n = 1), (B) metastatic tissues (colon metastatic to liver [n = 2] ovary [n = 1], lung [n = 1], pancreatic metastatic to liver [n = 1]; local nodal metastases from breast [n = 4], lung [n = 1], ovarian [n = 2]) (C) tumors from pre-treated patients (colorectal treated with oxaliplatin and bevacizumab [n = 1], breast treated with cyclophosphamide, methotrexate, fluorouracil [n = 2], breast treated with radiation, taxotere, cytoxan, taxol, (4 cycles each) [n = 1], NSCL treated with radiation [n = 2], ovarian treated with carboplatin and taxol [n = 2]) and (D) cell-line controls.

A wide range of cell-surface expression of EGFR, c-Met, and IGF-1R was observed among the tumor tissues that were analyzed, whereas in no case were significant levels of EpoR detectable on the cell-surface of the tumor cells from each of these tissues that were above the negative control cell-line or isotype control (Figs. 6 and 7). Significant levels of EpoR were not detected in the small cohorts of tumor tissues from metastatic tissues or from patients that had been previously treated with chemotherapy (Figs. 7B and 7C). The lack of EpoR expression suggested that EpoR was not induced in tumor cells during disease progression and was also not induced in response to treatment.

As previously mentioned, cell-lines (HT29 and UT-7/Epo) were processed in parallel as controls for cell-surface receptor expression with the analysis of every tumor tissue. In the analyses of all tumors described above, IGF-1R, EGFR, c-Met (using HT29 cells), and EpoR (UT-7/Epo cells) cell-surface expression were consistently demonstrated in the appropriate control cell-line (Fig. 7D).

Analysis of EpoR protein expression was also evaluated by Western blot analysis of 30 tumor tissues lysates from the breast cohort, including 6 Her2 positive tissues. Her2 status was assessed by a qualified pathologist for all breast tumor tissues. Consistent with reported Her2 prevalence [35], 6 of the 34 breast tumor tissues (same tissue samples used in Fig. 6) were Her2 positive as determined by IHC using the Dako Herceptest scoring system (25% tumor cells stain 2+ or 3+) using formalin fixed tissue collected from the tumor biopsy material (See S2 Table for additional details on Her2 status).

**Fig 8:**
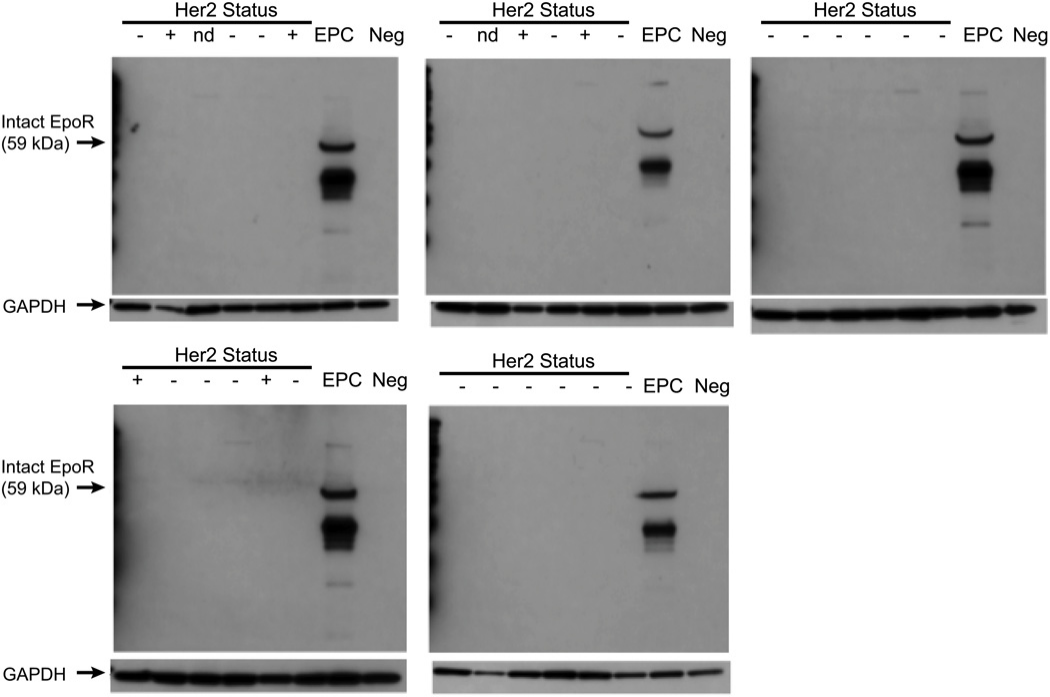
Analysis of EpoR protein expression in breast tumor tissues. Lysates prepared from an aliquot of tumor tissue from 30 patients from the cohort of breast tumor tissues that were analyzed by flow cytometry. Western blot analysis of EpoR expression was performed using the EpoR specific monoclonal A82. Erythroid progenitors (EPC) obtained following 8 days of differentiation *in vitro* from human bone marrow isolates cultured in the presence of IL-6, SCF, and rHuEpo were used as positive controls. GAPDH was used as a loading control.

Western blot analysis was conducted with the EpoR-specific A82 antibody (Amgen, Inc) that was previously shown to be suitable for Western Blot analysis [11]. This method also differed in that total EpoR (intracellular and surface) was examined. No EpoR protein was detectable using this method in any of the breast tumor tissues analyzed (Fig. 8). Lysates were also prepared from EPCs that had been differentiated from human bone marrow as a biologically relevant positive control. Expression of the mature EpoR protein was readily detectable (additional bands correspond to intracellular proteolytic fragments of EpoR as demonstrated by Mass Spectrometry protein sequencing [11]).

**Fig 9:**
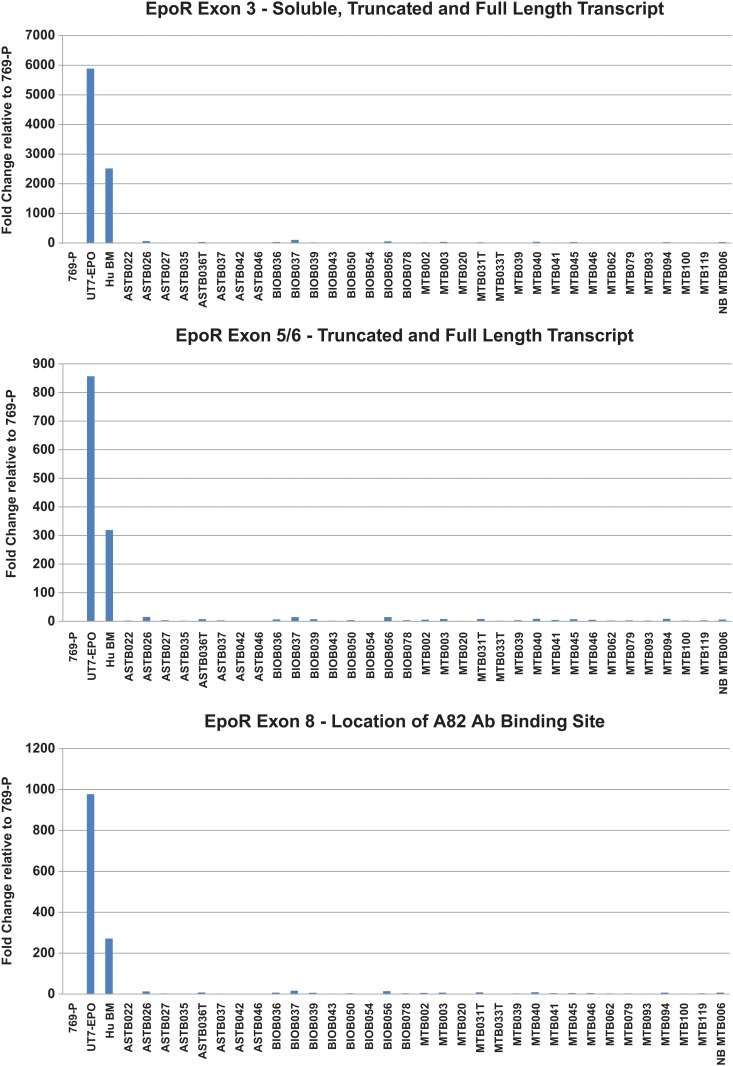
Analysis of *EpoR* mRNA expression in breast tumor tissues. Lysates were prepared from the tumor tissue of patients included in the cohort of breast tumor tissues analyzed by flow cytometry. Relative RNA abundance was measured using a TaqMan assay that amplified soluble, truncated, and full length *EpoR*. Data is expressed as a fold change relative to 769-P (*EpoR* negative control) and average reference gene abundance (*ACTB* and *GAPDH*); fold change = 2^-ddCq^. (A) *EpoR* exon 3—soluble, truncated, and full length transcript. (B) *EpoR* exon 5/6—truncated and full length transcript. (C) *EpoR* exon 8—location of A82 antibody binding site.

Analysis of *EpoR* mRNA expression levels was also conducted. *EpoR* mRNA was largely undetectable in the breast tumor tissues analyzed but readily detectable in the control UT7/Epo cell-line and in human bone marrow cells (Fig. 9). Taken together, these data support the conclusions derived from flow cytometry-based analysis of EpoR in that no expression or function was detectable in tumor tissues (Figs. 6 and 7) but EpoR expression and function was observed in EPCs at this stage of differentiation (Fig. 1).

## Discussion

Although early literature was inconsistent with functional EpoR expression in non-hematopoietic cells, including tumor cells and cell-lines [20, 23], safety signals reported in some recent clinical studies re-ignited interest in this question [36].

Eight of sixty controlled ESA trials reported increased mortality and/or disease progression with ESA use in the oncology setting and are included in the ESA product labeling information [31, 37]. Many controlled ESA oncology trials have not reported safety signals [7–9, 38–40], and differences in study design exist between these and studies that have reported mortality and progression safety signals [10]. In addition, a meta-analysis of 26 studies indicated that ESA use did not significantly affect disease progression [41].

Publications have since emerged suggesting functional EpoR is expressed in human tumors and human tumor cell-lines [10, 42]. They have formed the basis of an “EpoR tumor stimulation hypothesis” [36, 42]. Several potential issues have been identified for those reports that may confound their conclusions. Many used preparations of polyclonal anti-peptide antibodies in IHC and flow cytometry assays [23], assays for which those antibodies had not been validated [11, 21, 43, 44]. Other studies reported *EpoR* mRNA expression but without examining EpoR protein and function [22]. Functional studies with ESAs on tumor cell-lines were also conflicting and difficult to interpret, in part because they lacked appropriate positive and negative controls [12, 23, 28] to detect false-negative or false-positive effects.

*In vivo* Epo antagonism studies have reported that the blockade of Epo:EpoR inhibited tumor growth [45–47]. However, these results are inconsistent with *in vitro* findings that showed the same cell-lines had little/no EpoR expressed and had no detectable *in vitro* response when treated with ESAs, although it is possible that Epo in combination with other local and systemic growth factors may have an effect on tumor growth. In 31 different *in vivo* xenograft studies, no effect of Epo on tumor cell growth was observed [20].

Cytoprotection studies have also been conducted to assess whether ESAs have non-hematopoietic effects. In a number of animal studies, ESAs were reported to enhance angiogenesis after injury [48–58]. However, the results from these studies may be related to RBC increases, such as enhanced oxygen delivery or changes in ferrokinetics [59]. In other *in vivo* studies, ESAs did not provide non-hematopoietic protective effects [60–62] and the reported cytoprotective effects have generally not correlated with a clinical benefit in humans [11, 41, 63–73].

Most recently, more sensitive and specific reagents, controls and targeted protocols have been generated [11] and the question of EpoR protein expression and function on tumor cell-lines [27, 28] and other cell types [12, 19] has been re-examined [20]. Consistent with the earliest literature, those analyses have demonstrated that functional EpoR expression is essentially restricted to erythroid cells.

This study was designed to answer two questions using disaggregated tumor cells isolated directly from patient samples and employing rigorous protocols and controls: (i) do freshly isolated human tumor cells demonstrate Epo-induced signaling and (ii) do they express detectable EpoR (surface or intracellular, independent of the first question). No functional response to rHuEpo was detected in freshly-derived primary tumor cell populations. In addition, according to two different criteria, flow cytometry and Western blotting, no EpoR was detected in any tumor sample. It is possible that EpoR is expressed at levels below the threshold of detection for these assays or that an as yet unidentified receptor mediates “EpoR-like” pathway activation. However, the lack of a measurable pathway response to rHuEpo stimulation suggests that if EpoR was expressed at low levels, it was insufficient to drive a meaningful biologic response as defined by MAPK, PI3K, and pSTAT5 signaling.

Controls using validated phospho-specific and anti-EpoR antibodies, demonstrated assay sensitivity using Epo driven pathway induction of pSTAT5 and detectable expression of EpoR in differentiating erythroid cells. These studies demonstrated rHuEpo-dependent, functional EpoR early in differentiation of the culture when levels of cell-surface EpoR are known to be low. The late-stage erythroid cells have relatively higher levels of EpoR (approximately 800 to 1000 receptors on the cell-surface) [74–76] and these levels were readily detected with the reagents used. Additionally, viable epithelial tumor cells were specifically examined, and other cell-surface tumor growth factor receptors (EGFR, IGF-1R, and c-Met) were shown to remain functional under the conditions employed. Addition of a cocktail of known tumor growth factors to tumor cells uniformly demonstrated the activation of at least one of the key pathways that were interrogated. This further demonstrates that the methodology was sensitive and could detect meaningful responses in tumor cells. These pathways included the RAS/RAF/ERK and PI3K/AKT pathways that are essential for proliferation, survival, and metastatic spread of tumor cells.

Although we have attempted to perform these studies in a rigorous fashion, several possible caveats exist: (i) a very rare tumor cell subpopulation (ie, less than the assay sensitivity of ~2%) may express functional EpoR, albeit with questionable clinical relevance, (ii) the incidence of tumors with EpoR function may be too infrequent to have been detected in this study, and (iii) the actual receptor responding to rHuEpo is unique and does not utilize PI3K/AKT, MAPK, or STAT5 for signaling. These scenarios would represent a substantial departure from current knowledge and would require a fundamentally new understanding of Epo biology in tumors, and thus be a substantial departure from the assumptions underlying the hypothesis that Epo is a “tumor growth factor”, and that EpoR is widely expressed and functional on epithelial tumors.

With several possible explanations for the lack of a positive result, we conclude that evidence of a rHuEpo-responsive phenotype in patient derived tumor cells does not exist. Furthermore, EpoR was not detectable on human tumor cells isolated directly from multiple epithelial tumor types. This demonstrated that rHuEpo did not act directly on these tumor cells to promote growth and survival pathway signaling. Finally, the approach presented here may have considerable utility to address other important questions in tumor-cell signaling.

## Supporting Information

S1 Appendix Supplemental materials and methods. (DOCX)

S1 Fig Hematoxylin and eosin (H&E) stain of representative breast cancer samples. Identification of malignant cells and overall tumor content was assessed by a qualified pathologist for all samples included in the study. H&E images (20X magnification) from two breast cancer samples representing (A) low tumor content (6% tumor by area) and (B) high tumor content (80% tumor by area) are shown.
(EPS)

S2 Fig Induction of pERK and pAKT in matched normal and tumor cells. Nineteen matched normal and tumor samples were stimulated individually with EGF, HGF, and IGF1. FACS analysis was performed for AKT and ERK pathway utilization.
(EPS)

S3 Fig Gating strategy and representative histograms demonstrating Epo driven pSTAT5 induction in bone marrow derived eyrthroid precursors across dose and time. (EPS)

S4 Fig Representative histograms in UT7/ Epo control line demonstrating induction of (A) pAKT, (B) pERK, and (C) pSTAT5. (EPS)

S5 Fig Analysis of effect of dispase on expression and function of EpoR in UT-7/Epo. Disaggregation of tumor tissues to obtain single-cell populations employed proteolytic enzymes that did not compromise analysis of expression and/or function of cell-surface receptors. Dispase enzyme was used since it is selective for components of the extra-cellular matrix. While it was possible that dispase could reduce levels of cell-surface receptors including EpoR (if it were expressed) as well as IGF-1R, EGFR, and c-Met, this was not observed. To optimize enzyme digestion conditions to examine this possibility, EpoR cell-surface expression and function were evaluated in the presence of a range of dispase concentrations in UT-7/Epo cells. (A) Relationship between cell-surface levels of EpoR and dispase concentration. EpoR levels were reported as a ratio of mean MFI values relative to the appropriate isotype control. (B) Following dispase digestion, cells were stimulated with rHuEpo at 1U/mL for 5 minutes. Stimulated cells were fixed and permeabilized. To analyze signaling pathways, treated cells were stained with antibodies that are specific for pAKT and pSTAT5. Levels of phosphorylation were expressed as the ratio of MFIs following addition to vehicle-treated cells. This demonstrates that dispase does not interfere with the sensitivity of cell-surface EpoR nor EpoR function.
(EPS)

S6 Fig Gating strategy and representative histograms showing pathway response to 5 minute stimulation. (A) Vehicle, 300 U/mL rHuEpo or growth factor cocktail for (B) pERK induction, (C) pAKT induction, (D) pSTAT5 induction. Red plus sign indicates a measurable stimulation response to rHuEpo or growth factor cocktail.
(EPS)

S7 Fig Time course experiment of pAKT, pSTAT5, and pERK in the UT-7/Epo cell-line. To analyze signaling pathways, cells were stimulated with growth factors, harvested at various time points, and analyzed by flow cytometry with antibodies specific for pAKT, pSTAT5, and pERK. Levels of phosphorylation were expressed as the ratio of MFIs following addition to vehicle-treated cells.
(EPS)

S8 Fig pSTAT3 is not activated in response to rHuEpo in primary tumor cell populations from human tumor tissues. (a) colorectal (n = 33), (b) breast (n = 27), (c) non-small cell lung (n = 34), (d) ovarian (n = 25). Levels are reported as a ratio of mean fluorescent intensity (MFI) values relative to the appropriate isotype control. (Note: the ratio to isotype was used to normalize data and does not serve as baseline measure; i.e., a ratio of 1 does not imply a lack of stimulation).
(EPS)

S9 Fig EpoR surface expression concordance between MAb307 (R&D Systems) and A82 (Amgen, Inc) across a panel of negative and receptor positive cell-lines. (A) Flow cytometry with Y-axis values expressed as a fold change above a matched isotype control. (B) Correlation of EpoR surface expression as measured by flow cytometry. Y-axis values represent MAb307. X-axis values represent A82. Data is expressed as a fold change above a matched isotype control for each antibody.
(EPS)

S1 Table Aneuploid distribution in tumor samples. (DOCX)

S2 Table Details of Her2 status by immunohistochemistry (IHC). (DOCX)
