# Supplementary material for: Functional EpoR Pathway Utilization Is Not Detected in Primary Tumor Cells Isolated from Human Breast, Non-Small Cell Lung, Colorectal, and Ovarian Tumor Tissues
Source: PLoS One. 2015 Mar 25;10(3):e0122149. doi: 10.1371/journal.pone.0122149 (PMC4373902; doi:10.1371/journal.pone.0122149)
Supplement: S1 Appendix — (DOCX) [file pone.0122149.s001.docx]

**SUPPLEMENTARY INFORMATION**

**Functional EpoR Pathway Utilization Is Not Detected in Primary Tumor Cells Isolated From Human Breast, Non-Small Cell Lung, Colorectal, and Ovarian Tumor Tissues**

Scott D. Patterson*, John M. Rossi, Katherine L. Paweletz, V. Dan Fitzpatrick,

C. Glenn Begley, Leigh Busse, Steve Elliott, and Ian McCaffery

***Corresponding Author:**

Email: [spatters@amgen.com](mailto:spatters@amgen.com) (SP)

**S1 Appendix**

**Supplemental Materials and Methods**

**Cell Culture**

Cell culture reagents were from Invitrogen (Carlsbad, CA). UT-7/Epo was maintained in Iscove’s Modified Dulbecco’s medium containing 10% (v/v) fetal bovine serum (FBS) in the presence of penicillin (100 U/mL), streptomycin (100 µg/mL), 2 mM L-glutamine (1xPSG), and 1U/ mL rHuEpo (Amgen, Inc). HT29 cells were maintained in McCoy’s 5a Modified Medium containing 10% FBS (v/v) with 1xPSG.

**Analysis of Cell-surface Receptors in Live Cells by Flow Cytometry**

Antibody cocktails used were (1) EpoR-PE (MAb307; R&D Systems), EpCAM-APC (BD Biosciences, San Jose, CA), and CD45-APC/Cy7 (BD Biosciences); (2) anti-EpoR isotype control (mouse IgG2b-PE; R&D Systems), EpCAM-APC, and CD45-APC/Cy7; (3) EGFR-FITC ([AbD Serotec](%20http://www.abdirect.com/), Raleigh, NC), IGF-1R-PE (R&D Systems), c-Met-AF405 (R&D Systems), EpCAM-APC, and CD45-APC/Cy7; (4) isotype controls for EGFR (Rat IgG2a-FITC; [AbD Serotec](%20http://www.abdirect.com/)), IGF-1R (mouse IgG1-PE; R&D Systems), and c-Met (goat IgG1-AF405; R&D Systems) and EpCAM-APC and CD45-APC/Cy7.

**Analysis of EpoR Protein Expression by Western Blot Analysis**

After transfer, membranes were washed with Tris-buffered saline/Tween-20® (TBS-T: 25mM Tris-HCl, pH 7.5, 150mM NaCl, 0.15% Tween-20® [v/v]), then blocked at room temperature for 1 hour with 5% (w/v) non-fat dry milk in TBS-T. Blocked membranes were incubated with 0.1ug/mL EpoR specific monoclonal antibody A82 in 2.5% non-fat dry milk (w/v) plus TBS-T buffer for 3 hours at room temperature. Blots were washed in TBS-T, incubated in 0.01 ug/mL anti–rabbit IgG horseradish peroxidase-linked secondary antibody (Jackson ImmunoResearch Laboratories, West Grove, PA) at room temperature for 1 hour, and then washed 3 times in TBS-T. For detection, membranes were incubated for 5 minutes with ECL Plus reagent (Invitrogen) and then exposed to Hyperfilm ECL x-ray film. Equivalent loading of lysates was confirmed, immunoblots were stripped using Western Blot Stripping buffer (Thermo Fisher Scientific, Rockford, IL), and probed with 1:1000 dilution anti-GAPDH (EMD Millipore, Billerica, MA).

#### Analysis of DNA Content by Flow Cytometry (Table S1)

Disaggregated tumor samples were fixed and permeabilized as previously described for DNA content assessment. Briefly, cells were stained for 1hr at room temperature with fluorochrome-conjugated antibodies specific for pan-cytokeratin (clone C11, alexa flour 647; Cell Signaling Technology), CD45 (clone HI30, APC-Cy7; BD Biosciences) and active Caspase-3 (alexa fluor 405; Cell Signaling Technology). Antibody stained cell pellets were resuspended with 500µL propidium iodide (PI) /RNase Staining Buffer (BD Biosciences) and allowed to incubate for 15 minutes at room temperature PER the manufacturer’s protocol. Stained samples were run on an LSR II (Becton Dickinson, Franklin Lakes, NJ) with 10^4^ events acquired. The analysis used a multi-step gating strategy for exclusion of apoptotic events (caspase 3 positive), debris and aggregates. Single viable cytokeratin positive or CD45 positive events were further analyzed for DNA content. ModFit *LT* software (Verity Software House, Topsham, ME) was utilized to assess DNA content and cell cycle. Within each tumor sample, CD45 positive infiltrate was used as an internal comparator of normal DNA content (i.e. diploid) relative to the cytokeratin positive fraction.

#### Comparison of Matched Normal/ Tumor Signaling Profiles (Figure S2)

Tumor and matched normal colon tissue were processed according to previously described methodology for single cell suspensions. Aqua Viability Dye-labeled cell suspensions were stimulated for 5 minutes with vehicle, epidermal growth factor (EGF: 100 ng/ml, Roche), hepatocyte growth factor (HGF: 200 ng/ml, R&D Systems) or insulin-like growth factor-1 (IGF-1: 100 ng/ml, R&D Systems) in aliquots of 10^6^ cells. Treated cells were fixed and permeabilized as previously described and stored at -20^o^C prior to analysis. FACS analysis was performed as previously described for AKT and ERK pathway utilization.
