# Supplementary material for: Functional EpoR Pathway Utilization Is Not Detected in Primary Tumor Cells Isolated from Human Breast, Non-Small Cell Lung, Colorectal, and Ovarian Tumor Tissues
Source: PLoS One. 2015 Mar 25;10(3):e0122149. doi: 10.1371/journal.pone.0122149 (PMC4373902; doi:10.1371/journal.pone.0122149)
Supplement: S1 Table — (DOCX) [file pone.0122149.s011.docx]

**Table S1.** Aneuploid Distribution in Tumor Samples

| **Aneuploidy Percentage** | **Count** | **Percentage of Tumors Counted (N = 135)** |
| --- | --- | --- |
| Number of tumor with ≥ 5% aneuploid cells | 123 | 91% |
| Number of tumor with ≥ 15% aneuploid cells | 101 | 75% |
| Number of tumor with ≥ 50% aneuploid cells | 68 | 50% |
| Number of tumor with ≥ 75% aneuploid cells | 34 | 25% |
| Median aneuploidy percentage (interquartile range) | 56% (42% to 75%) |  |
