# Supplementary material for: Functional EpoR Pathway Utilization Is Not Detected in Primary Tumor Cells Isolated from Human Breast, Non-Small Cell Lung, Colorectal, and Ovarian Tumor Tissues
Source: PLoS One. 2015 Mar 25;10(3):e0122149. doi: 10.1371/journal.pone.0122149 (PMC4373902; doi:10.1371/journal.pone.0122149)
Supplement: S2 Table — (DOCX) [file pone.0122149.s012.docx]

**Table S2.** Details of Her2 Status by Immunohistochemistry (IHC)

|  | **Her2 IHC (Membrane Staining)** | | | | **Percent Positive** | **Max SI** | **H-Score** | **HercepTest Criteria** | **A82 Western Blot** |
| --- | --- | --- | --- | --- | --- | --- | --- | --- | --- |
| **Sample ID** | **3+** | **2+** | **1+** | **0** |  |  |  |  |  |
| ASTB022 | 0 | 2 | 96 | 2 | 98 | 2+ | 100 | Negative | Yes |
| ASTB026 | 98 | 0 | 2 | 0 | 100 | 3+ | 296 | Positive | Yes |
| ASTB035 | 0 | 0 | 40 | 60 | 40 | 1+ | 40 | Negative | Yes |
| ASTB036 | 0 | 0 | 98 | 2 | 98 | 1+ | 98 | Negative | Yes |
| ASTB037 | 4 | 56 | 40 | 0 | 100 | 3+ | 164 | Positive | Yes |
| ASTB038 | 0 | 0 | 98 | 2 | 98 | 1+ | 98 | Negative | Yes |
| ASTB042 | 0 | 0 | 95 | 5 | 95 | 1+ | 95 | Negative | No |
| BIOB037 | 0 | 5 | 94 | 1 | 99 | 2+ | 104 | Negative | Yes |
| BIOB039 | 1 | 14 | 83 | 2 | 98 | 3+ | 114 | Positive | Yes |
| BIOB043 | 0 | 1 | 99 | 0 | 100 | 2+ | 101 | Negative | Yes |
| BIOB050 | 0 | 3 | 94 | 3 | 97 | 2+ | 100 | Negative | Yes |
| BIOB052 | 0 | 0 | 100 | 0 | 100 | 1+ | 100 | Negative | No |
| BIOB054 | 0 | 1 | 89 | 10 | 90 | 2+ | 91 | Negative | Yes |
| BIOB056 | 0 | 3 | 97 | 0 | 100 | 2+ | 103 | Negative | Yes |
| BIOB078 | 0 | 0 | 30 | 70 | 30 | 1+ | 30 | Negative | Yes |
| MTB001 | 5 | 5 | 89 | 1 | 99 | 3+ | 114 | Positive | Yes |
| MTB002 | 0 | 5 | 94 | 1 | 99 | 2+ | 104 | Negative | Yes |
| MTB003 | 0 | 2 | 93 | 5 | 95 | 2+ | 97 | Negative | Yes |
| MTB018 | 0 | 0 | 100 | 0 | 100 | 1+ | 100 | Negative | Yes |
| MTB020 | 0 | 0 | 50 | 50 | 50 | 1+ | 50 | Negative | Yes |
| MTB031 | 5 | 23 | 70 | 2 | 98 | 3+ | 131 | Positive | Yes |
| MTB033 | 0 | 1 | 95 | 4 | 96 | 2+ | 97 | Negative | Yes |
| MTB039 | 0 | 0 | 20 | 80 | 20 | 1+ | 20 | Negative | Yes |
| MTB040 | 0 | 2 | 96 | 2 | 98 | 2+ | 100 | Negative | Yes |
| MTB041 | 100 | 0 | 0 | 0 | 100 | 3+ | 300 | Positive | Yes |
| MTB042 | 95 | 0 | 5 | 0 | 100 | 3+ | 290 | Positive | No |
| MTB045 | 5 | 5 | 88 | 2 | 98 | 3+ | 113 | Positive | No |
| MTB046 | 0 | 0 | 95 | 5 | 95 | 1+ | 95 | Negative | No |
| MTB048 | - | - | - | - | - | - | - | Negative | Yes |
| MTB049 | 0 | 2 | 98 | 0 | 100 | 2+ | 102 | Negative | Yes |
| MTB062 | 0 | 0 | 95 | 5 | 95 | 1+ | 95 | Negative | Yes |
| MTB079 | 0 | 0 | 97 | 3 | 97 | 1+ | 97 | Negative | No |
| MTB093 | 0 | 0 | 10 | 90 | 10 | 1+ | 10 | Negative | Yes |
| MTB094 | 0 | 0 | 100 | 0 | 100 | 1+ | 100 | Negative | No |
| MTB119 | 0 | 0 | 100 | 0 | 100 | 1+ | 100 | Negative | Yes |
| NB MTB004 | 0 | 0 | 100 | 0 | 100 | 1+ | 100 | Negative | No |
| NB MTB005 | 0 | 1 | 92 | 7 | 93 | 2+ | 94 | Negative | Yes |
| NB MTB006 | 0 | 9 | 61 | 30 | 70 | 2+ | 79 | Negative | Yes |
